# Imaging zinc speciation in the mouse hippocampus with µXANES Spectroscopic mapping

**DOI:** 10.1093/mtomcs/mfaf045

**Published:** 2026-01-02

**Authors:** Ashley L Hollings, Meg Willans, Virginie Lam, Ryu Takechi, John C L Mamo, Valerie Mitchell, Martin D de Jonge, Daryl L Howard, Gaewyn Ellison, Mark J Hackett

**Affiliations:** School of Molecular and Life Sciences, Curtin University, Perth, WA 6845, Australia; Curtin Medical Research Institute, Curtin University, Perth, WA 6102, Australia; School of Molecular and Life Sciences, Curtin University, Perth, WA 6845, Australia; Curtin Medical Research Institute, Curtin University, Perth, WA 6102, Australia; Curtin Medical Research Institute, Curtin University, Perth, WA 6102, Australia; School of Population Health, Faculty of Health Sciences, Curtin University, Perth, WA 6102, Australia; Perron Institute for Neurological and Translational Science, Perth, Australia; Curtin Medical Research Institute, Curtin University, Perth, WA 6102, Australia; School of Population Health, Faculty of Health Sciences, Curtin University, Perth, WA 6102, Australia; Perron Institute for Neurological and Translational Science, Perth, Australia; Curtin Medical Research Institute, Curtin University, Perth, WA 6102, Australia; School of Population Health, Faculty of Health Sciences, Curtin University, Perth, WA 6102, Australia; Perron Institute for Neurological and Translational Science, Perth, Australia; Australian Synchrotron, ANSTO Melbourne, 800 Blackburn Road, Clayton, VIC 3168, Australia; Australian Synchrotron, ANSTO Melbourne, 800 Blackburn Road, Clayton, VIC 3168, Australia; Australian Synchrotron, ANSTO Melbourne, 800 Blackburn Road, Clayton, VIC 3168, Australia; School of Molecular and Life Sciences, Curtin University, Perth, WA 6845, Australia; Curtin Medical Research Institute, Curtin University, Perth, WA 6102, Australia; School of Molecular and Life Sciences, Curtin University, Perth, WA 6845, Australia; Curtin Medical Research Institute, Curtin University, Perth, WA 6102, Australia

**Keywords:** metallomics, neuroimaging, brain, synchrotron, memory, dementia, zincergic, labile, mossy fibres

## Abstract

Zinc ions (Zn^2+^) are the second most abundant trace metal ion in the brain of rodents and primates, often serving functions as a structure-stabilizing element or catalytic role. There is an additional pool of Zn^2+^, ∼15% of total brain Zn^2+^, which exists in a labile chemical form in a specific subset of glutamatergic neurons (‘zinergic’ or ‘zincergic’ neurons). The labile pool of Zn^2+^ is now well established to be critical for healthy memory function, with disturbance to the labile Zn^2+^ pool implicated in diminished memory performance during the ageing process or neurodegeneration. The chemical form of Zn^2+^ in the labile Zn^2+^ pool has however, remained unknown, largely due to the difficulty of imaging metal speciation for ‘spectroscopically silent’ metals such as Zn^2+^. In this study, we have developed X-ray absorption near edge structure (XANES) spectroscopic protocols to enable chemically specific imaging of Zn^2+^ speciation in murine brain (hippocampal) tissue. The protocols capitalise on the unique sensitivity of the XANES spectral region to metal ion coordination environment, enabling a direct *in situ* measurement of metal speciation. Key findings of our method development are characterisation of the effects of sample preparation on metal speciation, and revelation that Zn^2+^ coordination with histidine is likely to be the dominant coordination environment of the labile Zn^2+^ pool in the murine hippocampus.

## Graphical Abstract

**Graphical Abstract:**
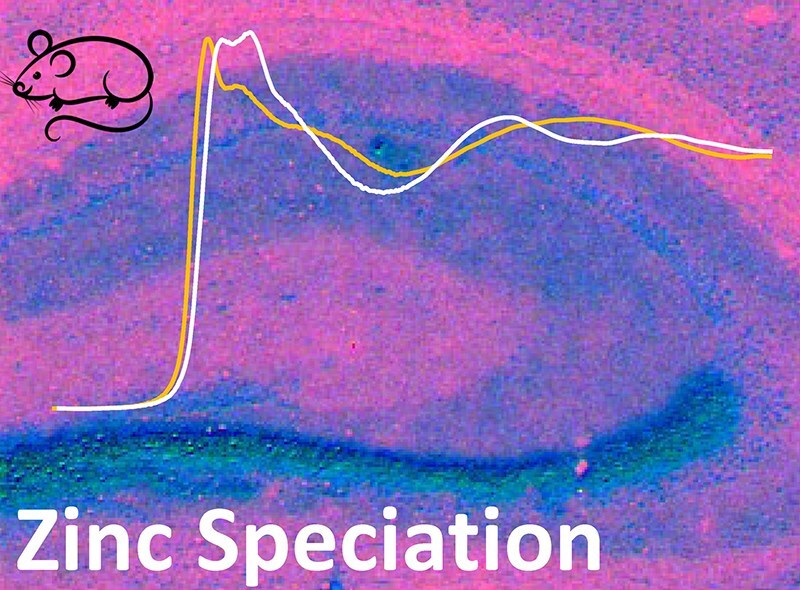

## Introduction

Zinc ions (Zn^2+^) are the second most abundant trace metal ion in the brain of rodents and primates, occurring at average concentrations of several hundred µM [1–3]. Throughout the central nervous system (CNS) Zn serves many biological functions, such as a structure-stabilizing element in proteins (i.e. Zn finger proteins, Cu-Zn super oxide dismutase); or catalytic roles at the active site of enzymes e.g. carbonic anhydrase, carboxy peptidase, alcohol dehydrogenase, alkaline phosphatase, β-lactamase, phospholipase C, to name a few [4–7]. Zn^2+^ has a valence electron configuration of Ar[4s0 3d10] and therefore, exists in one oxidation state in biological systems (+2), but still displays a diverse range of coordination chemistry through bonding to O, N, or S containing ligands [4–8]. Zn^2+^ generally prefers tetrahedral coordination geometry in proteins and enzymes, however, coordination with octahedral, distorted octahedral, and bipyramidal geometries are found [4–8], and often one ligand is loosely coordinated/labile [4–8]. For Zn^2+^ (and metal ions in general), the coordination environment (oxidation state, geometry, ligands) heavily influences the lability of metal ions, which has been leveraged by biological systems to control function and activity [9, 10].

Separate from incorporation within proteins and enzymes, ∼15% of Zn^2+^ within the CNS is in a labile form (sometimes referred to as the ‘free’ Zn^2+^ pool)[1–3]. A specific population of neurons, notably a subset of glutamatergic neurons (sometimes called ‘zinergic’ or ‘zincergic’ neurons) [3], are highly enriched in labile Zn, with local concentrations reported to reach several mM [1–3]. Sub-regions of the brain containing such zincergic neurons display highly localised concentrations of labile Zn^2+^, and include the olfactory bulb, layers III and V of the cortex, and the hippocampus (especially the hippocampal hilus and CA3 subiculum molecular layer or ‘mossy fibre layer’) [1, 11, 12].

Labile Zn^2+^ in the CNS, particularly in the hippocampus, is well established to play a pivotal role in memory function and synaptic plasticity [13–15]. There is substantial evidence from both preclinical animal models and human clinical data that demonstrate depletion of labile hippocampal Zn^2+^ correlates with diminished memory function [15–20]. Restoration of hippocampal Zn^2+^ following Zn^2+^ depletion restores memory function [15, 19, 21]. Despite the known relationship between hippocampal Zn^2+^, memory, and synaptic plasticity, the exact chemical form of labile Zn^2+^ and how chemical structure enables the biochemical and physiological pathways that drive memory function remain unknown. Increased understanding of the coordination environment of the labile Zn^2+^ pool may help reveal the biochemical pathways through which the labile Zn^2+^ becomes dysregulated during ageing and neurodegenerative disease leading to poorer memory function. In addition, knowledge of the coordination environment of labile Zn^2+^ may help inform therapeutic developments aimed at restoring this pool to improve memory function.

Studying Zn^2+^ speciation in biology has long been an analytical challenge, as Zn^2+^ is often referred to as a ‘spectroscopically silent’ element. As opposed to other commonly studied transition metal ions (Fe and Cu), the major isotope of Zn (^64^ Zn) does not have nuclear spin and also contains a full 3d electron shell and Zn^2+^ is therefore not detectable with NMR or EPR [22]. To study brain Zn^2+^ homeostasis many researchers have used histochemical stains or fluorescent Zn^2+^ sensors to detect labile Zn^2+^. Early protocols developed by Timm and Danscher detected the labile Zn^2+^ pool via histochemical precipitation as a sulfide or selenide followed by auto-metallographic enhancement [1, 11, 12, 23–28]. Unfortunately, there are a range of limitations with histochemical methods. Namely, histochemical Zn^2+^ detection often requires perfusion of living animals with the relevant sulfide or selenide which is technically challenging, poses animal welfare risk if concomitant anaesthesia fails, can be prone to variable results, and is not-quantitative [29]. Further, histochemical methods, although sensitive to labile Zn^2+^, do not reveal any information regarding the speciation of Zn^2+^ (other than it is in a labile form), and false-positive staining from histochemical reactions with other metal ions can occur.

More recently, a range of fluorescent Zn^2+^ chelators (sensors) (e.g. Zn-TSQ, Zinquin, Newport green) have been developed [10, 30–33]. Although incredibly useful to study labile Zn^2+^ pools *in vitro* within cell culture or within organotypic living tissue slices, fluorescent Zn^2+^ sensors have had limited use to *in vivo* application or *ex vivo* application to tissue sections obtained from animal models. One reason behind the inability to apply fluorescent Zn^2+^ sensors *ex vivo* to brain tissue sections is the mobile nature of labile Zn^2+^, with the fixation and/or staining process itself redistributing hippocampal Zn^2+^ [34]. Another limitation of fluorescent sensor detection, similar to histochemical methods, is that the technique does not reveal information on the chemical form of Zn^2+^ being detected (other than being labile), and cross reactivity with other divalent cations, especially Ca^2+^, is possible. Despite these challenges, histochemical methods and fluorescent sensors have revealed a great volume of vital information on labile Zn^2+^ within the CNS during brain development, brain ageing and neurodegeneration (e.g. Alzheimers [35, 36], stroke [37], epilepsy [38, 39]), including the initial discovery of the labile Zn pool itself [1, 11, 12, 40, 41].

Another approach to study brain Zn^2+^ homeostasis, although less commonly used, is Zn^2+^ sensitive magnetic resonance imaging (MRI) contrast agents. Indeed, development of Zn^2+^ sensitive MRI contrast agents is emerging as a promising tool to study labile Zn^2+^*in vivo* [42–44]. The spatial resolution of MRI however, is limited to hundreds of microns, at best, and thus it is not compatible with imaging labile Zn^2+^ at the cellular level. A technique that is capable of direct measurement of the chemical form of Zn^2+^ in *ex vivo* brain tissue (and more broadly in all cells and tissues) is therefore a much needed analytical tool.

One technique that offers promise for *in situ* analysis of metal ion coordination environment in complex biological samples is micro X-ray absorption near-edge structure (µXANES) spectroscopy. µXANES enables the study of metal speciation directly in *ex vivo* tissue sections, without the need for chemical fixation, staining, or addition of contrast agents [10, 32, 45–47]. For 1^st^ row d block metals, such as Zn, K-edge µXANES spectroscopy probes the excitation of a 1 s core electron to unoccupied molecular orbitals with 4p character, which provides an excellent spectroscopic fingerprint of oxidation state and coordination environment (e.g. ligand type and coordination geometry) [22, 48, 49]. µXANES spectroscopy at the K-edge has found important applications for *in situ* analysis of Fe and Cu speciation in *C. elegans models* [50, 51], and *D. melanogaster* models [52], with applications of K- and L-edge used to image Fe and Cu oxidation state in brain tissue [53–57]. µXANES spectroscopic mapping protocols have also previously been developed to image Cu speciation within environmental samples [58] and sulfur speciation in plant [59] and brain tissue [60], however to date, chemically specific imaging of Zn speciation in cells and tissues has not been achieved.

Despite the lack of established µXANES spectroscopic mapping protocols that enable chemically specific imaging of Zn speciation in biological systems, XANES spectroscopy has long been recognised as a useful probe of Zn^2+^ coordination environment [22, 48, 61]. Studies that have used µXANES spectroscopy to characterise Zn^2+^ speciation in biological systems include applications to plant tissue [62–65], bacteria [66], macrophages [67], and our own studies of Zn^2+^ speciation in murine brain tissue [68]. A key component to using µXANES spectroscopy to determine Zn^2+^ speciation in complex biological systems is the development of appropriate spectral libraries, with recent studies developing detailed XANES spectroscopic measurements of standard solutions [63, 65, 68, 69], in addition to high energy resolution fluorescence detection (HERFD) analyses and complementary fluorescence emission analysis [49, 66]. Another important consideration for all studies attempting to investigate metal ion speciation in *ex vivo* tissue sections is the effect of sample preparation, which can have a large impact redistributing or changing the speciation of metal ions between the *in vivo* and *ex vivo* state [34, 68, 70,71].

In this study herein we demonstrate, to the best of our knowledge, the first ever application of µXANES spectroscopy to directly image multiple chemical forms of Zn^2+^ in *ex vivo* brain tissue sections. Our results showcase the immense analytical potential of µXANES spectroscopy to image coordination chemistry and metal ion speciation *in situ* within biological tissues. We also demonstrate key alterations to Zn^2+^ speciation that can occur during sample preparation, and our results highlight that analysis of frozen hydrated tissue sections under cryogenic conditions is essential to preserve the *in vivo* state. The chemically specific Zn^2+^ images obtained by µXANES spectroscopy reveal striking differential patterns of Zn^2+^ speciation within murine hippocampal tissue. Significantly, the ability to image Zn^2+^ speciation *in situ* has revealed strong evidence supporting that the labile Zn^2+^ pool found within the hippocampal mossy fibres exists coordinated through N donors of histidine ligands, with no spectroscopic evidence of ‘free’ or hexaaqua Zn observed.

## Methods

### Brain tissue sample preparation

#### Animal models

Six-month-old senescence-accelerated mice prone 8 (SAMP8) mice were used in this study (n = 3 biological replicates). All animal work was conducted in accordance with Curtin University Animal Ethics Guidelines under an approved Curtin animal ethics protocol (AEC-2014-27). Mice were housed in standard cages in a temperature-controlled room (21°C) with 12 hr light/dark cycle and ab libitum access to food (standard rodent chow) and water.

#### Tissue preparation and sample transport

To preserve Zn speciation as close as possible to the *in vivo* condition, animals were anaesthetized with isoflurane and humanely sacrificed via cervical dislocation and the brain tissue rapidly dissected into sagittal hemispheres, which were immediately plunge frozen in liquid nitrogen cooled isopentane [70, 72, 73]. Two weeks before µXANES spectroscopic analysis at the X-ray fluorescence microscopy (XFM) beamline at the Australian Synchrotron, 10-μm-thick coronal brain sections were cut from the frozen brains at a Bregma location of -1.5 mm to -2 mm using a cryo-microtome operating between -16 to -18 °C and melted onto a silicon nitride substrate (Melbourne Centre for Nanofabrication, 10 × 10 mm^2^ 200-µm-thick silicon frame, and a 5 × 5 mm^2^ 1000-nm-thick silicon nitride membrane). Two sets of tissue sections were prepared: frozen-hydrated or dehydrated air-dried. For frozen-hydrated tissue sections, after the tissue section was melted onto the silicon nitride membrane the tissues were immediately transferred into a -80 °C freezer. One week before the XFM analysis, the frozen-hydrated samples were shipped on dry ice to the Australian Synchrotron (< 48 hours travel on dry ice). Samples were immediately returned to a -80 °C freezer following arrival at the Australian Synchrotron. At the time of µXANES analysis, frozen-hydrated samples were taken from the—80 °C freezer and placed in dry ice and transported to the XFM beamline. At the XFM beamline the frozen-hydrated samples were mounted directly from dry ice into the liquid nitrogen cryostream for analysis. For dehydrated air-dried samples, following melting of the tissue section onto the silicon nitride membrane, samples were allowed to air-dry at ambient room temperature, and were then stored at room temperature in the dark in the presence of desiccant (CaSO_4_) until required for µXANES analysis (which was undertaken at room temperature).

### Bulk XANES spectral collection and analysis of standard solutions

#### Preparation of standard solutions

A range of standard solutions were prepared to expand our previous spectral library[68] to include Zn^2+^ in the presence of excess acetate, lactate, citrate, EDTA, aspartate, pyruvate, or Br^−^ (from KBr). In addition, data was collected from an aqueous solution of metallothionein. The aqueous standard solutions were prepared as described previously (1 mM Zn^2+^ prepared from Zn(NO_3_)_2_ in the presence of 10 mM ligand)[68]. The standard solutions collected previously which were also used for linear combination fitting in this study included Zn^2+^ + excess: histidine (with or without glycerol glassing agent), cysteine, glutamate, phosphate, Cl^−^ and hexaaqua complexes at pH 7 and 13 [68].

#### Collection of bulk XAS (XANES and EXAFS) of standard solutions

Data acquisition was undertaken at the XAS beamline at the Australian Synchrotron (ANSTO), as described previously (1.9T wiggler, Si(111) double crystal monochromator, harmonic rejection with Si and Rh mirrors, beam size at samples ∼ 1 × 0.5 mm) [68]. Spectra were collected using Kα emission, with samples mounted at 45 degrees to the incident X-ray beam in a liquid helium-cooled cryostat (12—15 K) using a Canberra liquid nitrogen-cooled 100 pixel monolithic solid state Ge detector. Energy calibration was performed using the transmission spectrum of Zn metallic foil recorded with downstream ion chambers (calibrated to the first inflection peak of Zn metal foil at 9660.7 eV) [68]. Glassing agents were not used for collection of any standard solution spectra with the exception of Zn^2+^ + excess histidine (where spectra from two standard solutions were collected, one with and one without addition of a glycerol glassing agent). XANES spectra were recorded across the K-edge white line features from 9650 to 9700 eV at 0.3 eV steps (500 ms dwell per data point). EXAFS spectra were recorded across the K-edge from 9639—9709 eV at 0.25 eV steps (1 s dwell per data point) and the post-edge region was recorded to a k value of 12 at 0.035 Å^−1^ steps (dwell increasing linearly from 1 to 4 s). Due to ice crystal diffraction artefacts at high k, spectra are presented to a k value of 10. Raw data was visualised using Sakura software v2.7 [74], to exclude data from detector pixels containing contributions from ice-diffraction, after which data was then exported in ASCII format and further analysed using EXAFSPAK (XANES spectral analysis) [75], and Larch (EXAFS spectral analysis) [76].

#### Processing and analysis of XANES spectra of standard solutions

Spectra were background corrected (polynomial background subtraction, calculated from pre-edge region) and normalised to a post-edge jump of 1 absorbance unit. Position and intensity of white line features of standard solutions are reported for background correction and normalised XANES spectra.

#### Analysis of EXAFS spectra from standard solutions

A smooth background function was subtracted using the AUTOBK algorithm [77]. Spectra were converted to k-space (k^3^-weighted), and Fourier transformed into R-space. Phase correction was not applied. EXAFS were simulated with FEFF8L *ab initio* calculations and fit to experimental spectra in R-space. S_0_^2^ was determined by setting N = 4 for the metallothionein spectrum and held constant for all other calculations. ΔE_0_, R, and σ^2^ were refined for the first shell of each spectrum (1-2.5 Å), except for the histidine solutions where further shells were fit (1-4 Å). Fit results were compared for varying N, and the best fit reported.

### µXANES Mapping of Zn^2+^ speciation in murine hippocampal tissue

#### µXANES spectroscopic mapping data collection (chemically specific Zn^2+^ imaging)

XANES-spectroscopic maps (‘XANES-stack’), were collected at the X-ray Fluorescence Microscopy beamline at the Australian Synchrotron [78]. The XANES stacks were collected across 125 incidence energies (1 ms dwell), from high energy to low energy: 9803—9723 eV (post-edge, 10 eV steps), 9723—9699 eV (post-edge 2 eV steps), 9699—9650 eV white line region, 0.5 eV steps), 9650—9630 eV (pre-edge region, 5 eV steps). The photo-flux, measured with an ion chamber immediately before the KB mirrors, is estimated at 8.8 × 10^9^ photons per second. Although 0.1 or 0.2 eV steps is more commonly used for white line features across the Zn K-edge when undertaking bulk XAS analysis, due to the substantially larger time requirements of spectroscopic mapping, 0.5 eV steps across the white line enable the experiments to be feasible within the realistic time frame (e.g. several hours of data collection). The process of XANES spectroscopic map data collection is shown in Schematic 1. Beamline energy calibration was performed as described for bulk XANES and EXAFS data collection (downstream ion chambers and Zn metal foil). Maps were collected with 2 µm steps and a micro-focussed X-ray beam (2 µm, 2-σ, using Kirkpatrick—Baez mirror pair). The X-ray fluorescence emission from the sample was recorded with the sample 45 degrees to the incident beam using a 3-element Vortex Si-drift detector. Maps of the Zn Kα-emission maps were reconstructed from the full emission spectra with GeoPIXE v6.6j (CSIRO, Australia) [79], as in our previous studies [80–82]. Air-dried dehydrated samples were analysed under ambient laboratory conditions, while frozen-hydrated samples were analysed under a liquid nitrogen cryo-jet at ∼120 K (-153°C). Frozen-hydrated samples were transported from the -80°C freezer to the XFM beamline on dry ice, mounted onto the sample holder using doubled-sided tape (while on dry ice), and then immediately transferred into the cryostream (< 30 seconds between transfer of sample from dry ice into the cryostream). The image ‘field of view’ (i.e. sample map area) had to be smaller for the cryostream sample compared to the air-dried sample, to ensure the entire sample stayed within the dimensions of cryostream—preventing sample thawing during measurement.

**Schematic 1.**
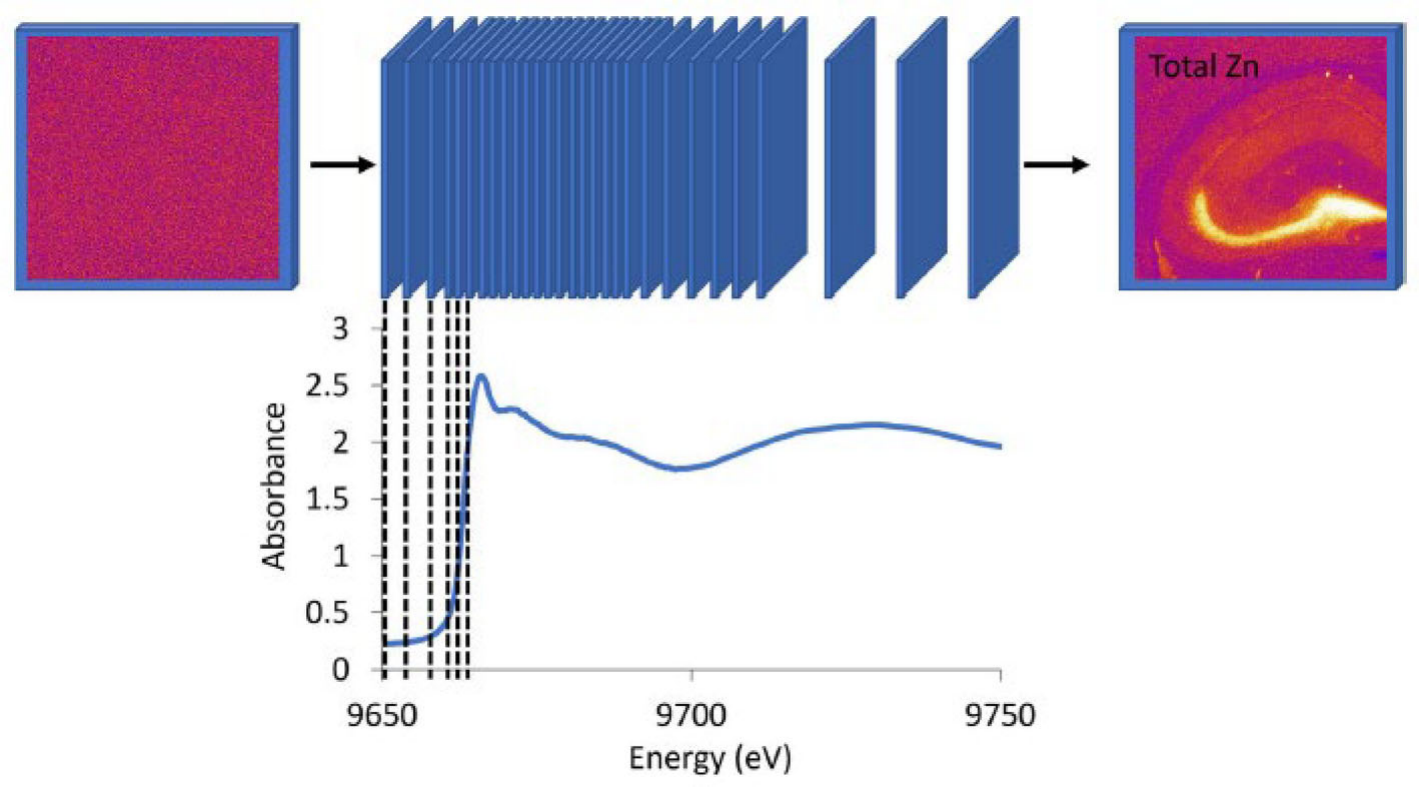
Schematic representation of the collection of Zn K_α_ fluorescence maps across a ‘Z-stack’ of incident X-ray excitation energies that span the Zn K-edge XANES spectrum.

#### µXANES mapping data analysis

**Figure 1.**
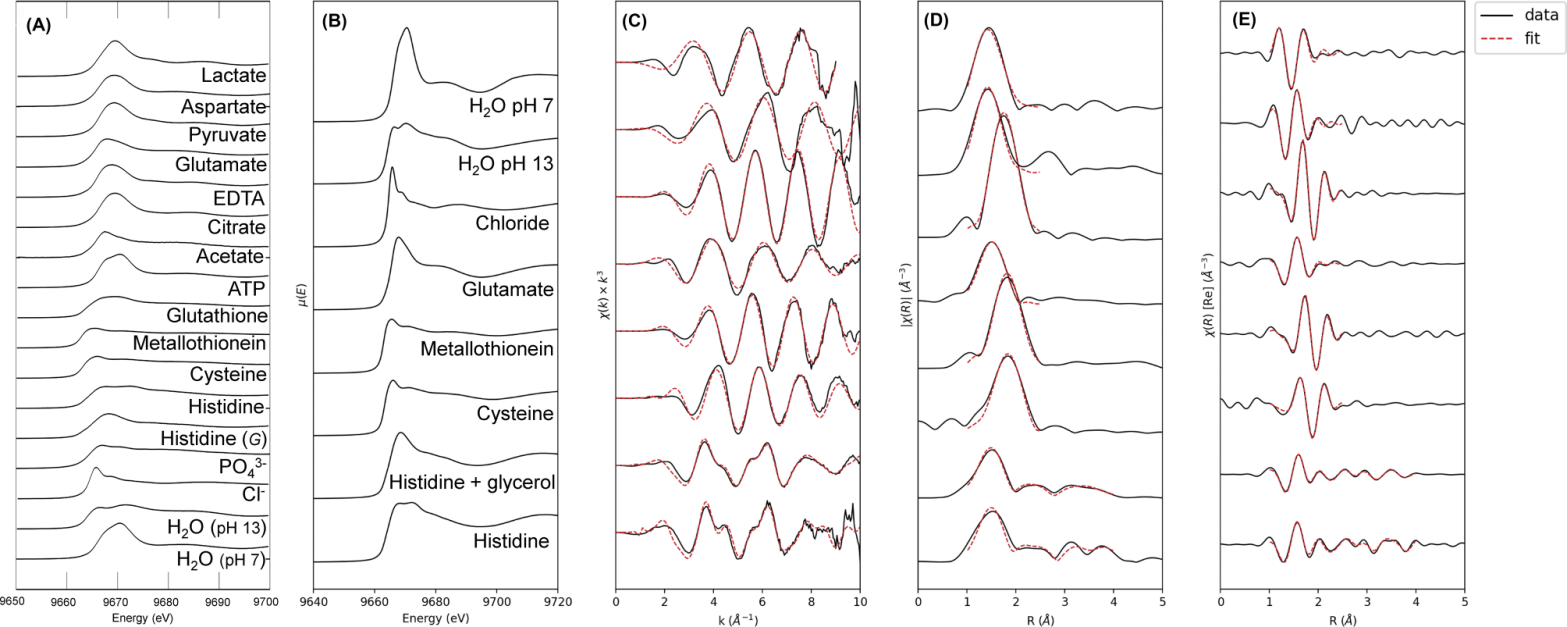
XANES and EXAFS spectral characterisation of standard solutions. (**A**) zn K-edge XANES spectra of standard solutions used for XANES spectral library. dash lines indicate position of lowest energy white line feature for a standard solution in the library (9664.9 eV—metallothionein, and 9669.7 eV—hexaaqua Zn^2+^ +pH 7). (**B**) XANES spectra of standard solutions that were subsequently analysed with EXAFS spectroscopy. (**C**) zn K-edge k^3^-weighted EXAFS spectra, (**D**) fourier transform magnitude, and (**E**) fourier transform real component with EXAFS fitting results for Zn^2+^ standard solutions. for the standard solution of Zn^2+^ in water at pH 13, a longer-range scattering peak is potentially observable, suggestive of a second shell zn—zn interaction. although any interpretation of bonding environment beyond the first coordination shell is difficult due to the limited k-range of this data set, modelling of a zn—zn interaction, possibly suggesting the presence of a small amount of zn(oh)_2_ precipitate in this sample, is shown in supporting information Fig. SI1.

Elemental maps of Zn fluorescence intensity were extracted as TIFF files from GeoPIXE and converted into a Z-stack (Z-dimension equating to incident energy) within ImageJ (v1.48). To improve spectral signal to noise 4×4 pixel binning was applied, to yield images with a 8×8 µm pixel size. Average Zn^2+^ µXANES spectra from specific regions of interest (ROIs) were generated in imageJ, for ROIs corresponding to hippocampal mossy fibre region and CA3 pyramidal cell layer, as described previously (representative examples of the ROIs are shown in Fig. 1) [68]. The average µXANES spectra from each ROI were further analysed using the EXAFSPAK suite of data analysis programs.[75] For chemically specific Zn^2+^ imaging, the Zn^2+^ µXANES spectra were normalised to a uniform Zn content and exported from ImageJ in a .tiff format, and then imported into MANTIS for initial exploration using principal component analysis, followed by image deconvolution and generation of chemically specific Zn^2+^ images. De-convolution of the µXANES maps was performed using single value decomposition (SVD) within MANTIS software [83], as previously described in the literature [83, 84]. The choice of model spectra (spectra of standard solutions) to use for SVD was informed following linear combination fitting of average µXANES spectra from specific regions of interest, as described below.

#### Linear combination fitting of average µXANES spectra from ROIs

Linear combination fitting of µXANES spectra from tissue ROIs, fit to spectra of standard solutions, was performed in EXAFSPAK (using ‘Datfit’), as previously described [68]. An extensive exploration of fitting combinations was trialled, fitting from 2—7 model spectra. Ultimately, for all samples it was determined that the average Zn^2+^ K-edge XANES spectrum from the ROIs, could be adequately fitted to a linear combination of 3 spectra, corresponding to standard solutions of Zn^2+^ + excess histidine, Zn^2+^ + excess phosphate and Zn^2+^ cysteine in aqueous solution. Addition of extra components into the fit, did not reduce the fit residuals, but it was noted that the XANES spectra of the standard solution for Zn^2+^ + excess cysteine could be substituted for metallothionein, yielding near identical results (and identical scientific conclusions, as shown in Supporting Information Fig. SI2). Across all fits, the fit residuals ranged from 0.0008 to 0.002, and the estimated standard deviation of each individual component across all the fits ranged from 0.025 to 0.075 (i.e. 2.5—7.5%).

#### Statistical analysis

Statistical analyses were performed using Graphpad Prism v8, and all data is presented as scatter plots. Testing for differences in Zn^2+^ speciation was undertaken for the following comparison: Zn^2+^ speciation in the CA3 mossy fibres in frozen-hydrated tissue vs air-dried tissue; Zn^2+^ speciation in the CA3 pyramidal neurons in frozen-hydrated tissue vs air-dried tissue; Zn^2+^ speciation in CA3 mossy fibres in frozen-hydrated tissue vs CA3 pyramidal neurons in frozen-hydrated tissue; Zn^2+^ speciation in CA3 mossy fibres in air-dried tissue vs CA3 pyramidal neurons in air-dried tissue. Each experimental group contained (*n* = 3 biological replicates), and statistical testing was performed using a Student’s *t*-test and 95% confidence interval. A paired *t*-test was used when comparing between ROIs (e.g. comparing Zn^2+^ speciation in air-dried tissue between the CA3 mossy fibres and CA3 pyramidal neuron layer). A non-paired *t*-test was used when comparing between sample preparation (e.g. comparing Zn^2+^ speciation in CA3 mossy fibres between air-dried and frozen-hydrated tissues).

## Results and discussion

### Characterisation of standard solution of Zn^2+^ coordination complexes—zinc XANES spectra are exquisitely sensitive to coordination environment

We have previously published our initial development of a Zn^2+^ K-edge XANES spectroscopic library for biological applications, using standard solutions of Zn^2+^ in the presence of excess biological ligand, [68] which is a similar approach to that used by others [62, 63, 65, 66, 68, 69]. In this study here, we have expanded on that spectral library to include additional standard solutions of Zn^2+^, and EXAFS characterisation of the biological relevant complexes of Zn^2+^ with excess cysteine or histidine in solution. The new additions to the spectral library include: Zn^2+^ in the presence of excess carboxylic acids (citrate, acetate, aspartate, lactate, pyruvate (Fig. 1A, Table 1); and Zn^2+^ metallothionein in aqueous solution (Fig. 1A, B, Table 1). The EXAFS characterisation is presented in Fig. 1C-E, and Table 2).

**Table 1.** Summary of ‘white line’ characteristic features in XANES spectra of Zn^2+^ standard solutions

Ligand (added in 10:1 excess to Zn^2+^)	Position of white line maximum intensity(eV)	White line maximum intensity	Notes
H_2_O (pH 7)	9669.7	2.39	Low energy shoulder at 9667.8 eV (2.10)
ATP	9669.7	2.30	Low energy shoulder at 9667.8 eV (2.08), closely resembles hexaaqua complex(Zn^2+^ in H_2_O at pH 7)
Citrate	9669.3	2.24	
Lactate	9668.8	2.37	
Pyruvate	9668.8	2.26	
Aspartate	9668.8	2.08	
Glutathione	9668.6	1.43	
EDTA	9668.3	2.15	
Histidine (*glycerol*)	9667.7	1.67	No high energy feature
Glutamate	9667.3	1.80	
Acetate	9667.2	1.71	
Histidine	9667.0	1.32	High energy feature at 9671.8 eV (1.35)
PO_4_^3−^	9666.5	1.61	
H_2_O (pH 13)	9665.9	1.24	High energy feature at 9670.9 eV (1.40)
Cysteine	9665.3	1.35	
Cl^−^	9665.0	1.90	
Metallothionein	9664.9	1.31	

**Table 2.** EXAFS curve-fitting results of Zn^2+^ standard solutions

Ligand	Shell	N	R	σ^2^	R-factor
Zn^2+^_(aq)_ + excess histidine	Zn-N	4	2.02(1)	0.008(1)	0.057
	Zn-C	8	3.01(4)	0.019(4)	
	Zn-N	8	3.87(5)	0.008(5)	
	Zn-N-C	15	4.32(4)	0.001(5)	
Zn^2+^_(aq)_ + excess histidine + glycerol	Zn-O	2	2.00(3)	0.006(4)	0.014
	Zn-N	2	2.06(9)	0.013(10)	
	Zn-C	4	2.96(2)	0.009(3)	
	Zn-N	4	3.89(4)	0.004(5)	
	Zn-N-C	8	4.31(4)	0.001(9)	
Zn^2+^_(aq)_ + excess cysteine	Zn-S	4	2.31(1)	0.010(1)	0.014
Zn^2+^_(aq)_ + excess metallothionein	Zn-S	4	2.34(1)	0.006(1)	0.009
Zn^2+^_(aq)_ + excess glutamate	Zn-O	4	2.00(1)	0.007(1)	0.013
Zn^2+^_(aq)_ + excess chloride	Zn-Cl	4	2.27(1)	0.004(1)	0.009
Zn^2+^_(aq)_ + H_2_O (pH = 13)	Zn-O	4	1.94(2)	0.002(1)	0.039
Zn^2+^_(aq)_ + H_2_O (pH = 7)	Zn-O	4	2.02(2)	0.002(1)	0.028
Zn^2+^_(aq)_ + H_2_O (pH = 7)^a^	Zn-O	6	2.02(2)	0.006(1)	0.042

a Alternative fit (N = 6) applied to the same spectrum with similar results, highlighting uncertainty in N in EXAFS curve fitting.

The XANES spectra presented in Fig. 1A show remarkable sensitivity to coordination environment as can be seen by the variation in the position, shape and intensity of the white line as a function of coordinating ligand (Table 1). Spectral features of note include the high white line intensity (absorbance values > 2 relative to a normalised edge-jump of 1) of Zn^2+^ in the presence of excess lactate, pyruvate, citrate, aspartate, and EDTA (Fig. 1A, Table 1), suggesting octahedral coordination. Interestingly, the white line intensity is reduced for Zn^2+^ in the presence of excess glutamate or acetate (Table 1), suggesting a lower coordination number or distorted coordination environment.

Many biological molecules have multiple sites for metal ion coordination, such as carboxylate group or thiol groups in cysteine. We have therefore, compared XANES spectra of Zn^2+^ in the presence of excess cysteine at neutral pH, and metallothionein (Fig. 1 A, B), which display minimal differences in the XANES region. The white line feature of both Zn^2+^ + excess cysteine standard solution and the metallothionein solution standard display reduced intensity and a shift to lower energies, relative to XANES spectra of either the hexaaqua Zn^2+^ complex or Zn^2+^ + excess citrate, indicating a lower coordination number of Zn^2+^ in metallothionein or Zn^2+^ in the presence of excess cysteine (e.g. consistent with a tetrahedral coordination geometry). The shift to lower energy of the white line feature associated with thiol coordination is consistent with coordination through less electronegative atoms such as S (relative to O). As a ‘fingerprinting’ tool, the strong reduction in white line intensity and shift to lower energy of the white line for tetrahedral thiol coordination of Zn^2+^, enables simple discrimination from Zn^2+^ coordination with organic acids. These interpretations are supported by EXAFS spectroscopic analysis of both the metallothionein standard solution, and the standard solution of Zn^2+^ + excess cysteine, which indicates for both a coordination number of 4, with S as the coordinating atom, with a Zn—S bond length of 2.31 (solution complex with excess cysteine) or 2.34 (aqueous metallothionein), which is consistent with the literature (Fig. 1C-E, Table 2) [66, 85–87]. Although Zn coordinates as a multi-metallic thiolate cluster in metallothionein, longer range scattering peaks (that would be consistent with a second shell Zn—Zn interaction) were not observed in the EXAFS spectra of either metallothionein or the Zn^2+^ + excess cysteine standard solution. However, we are careful not to over interpret this observation, due to the reduced k-range of the data collected and relatively low signal to noise levels.

Ourselves and others have previously reported that the XANES spectrum of Zn^2+^ + excess histidine shows a distinct doublet feature, differentiating Zn^2+^ histidine coordination from Zn^2+^ coordinated to either thiols or organic acids [66, 68]. Interestingly, XANES spectra of Zn^2+^ in the presence of excess histidine show pronounced spectroscopic differences depending on whether or not a glycerol glassing agent is added (Fig. 1A, B) [68]. These differences have been further characterised with EXAFS spectroscopy in this current study (Fig. 1C-E). The spectra both with and without added glycerol showed longer range peaks in the 2.5-4 Å range consistent with scattering contributions expected from coordination through imidazole N [66]. The multiple scattering contribution at ∼4 Å is reduced in the solution containing glycerol, suggesting mixed oxygen and imidazole coordination, and this is supported by the EXAFS fitting (Fig. 1C-E, Table 2).

### Principal component analysis provides an effective data reduction and visualisation tool for initial exploration of XANES spectroscopic maps

**Figure 2.**
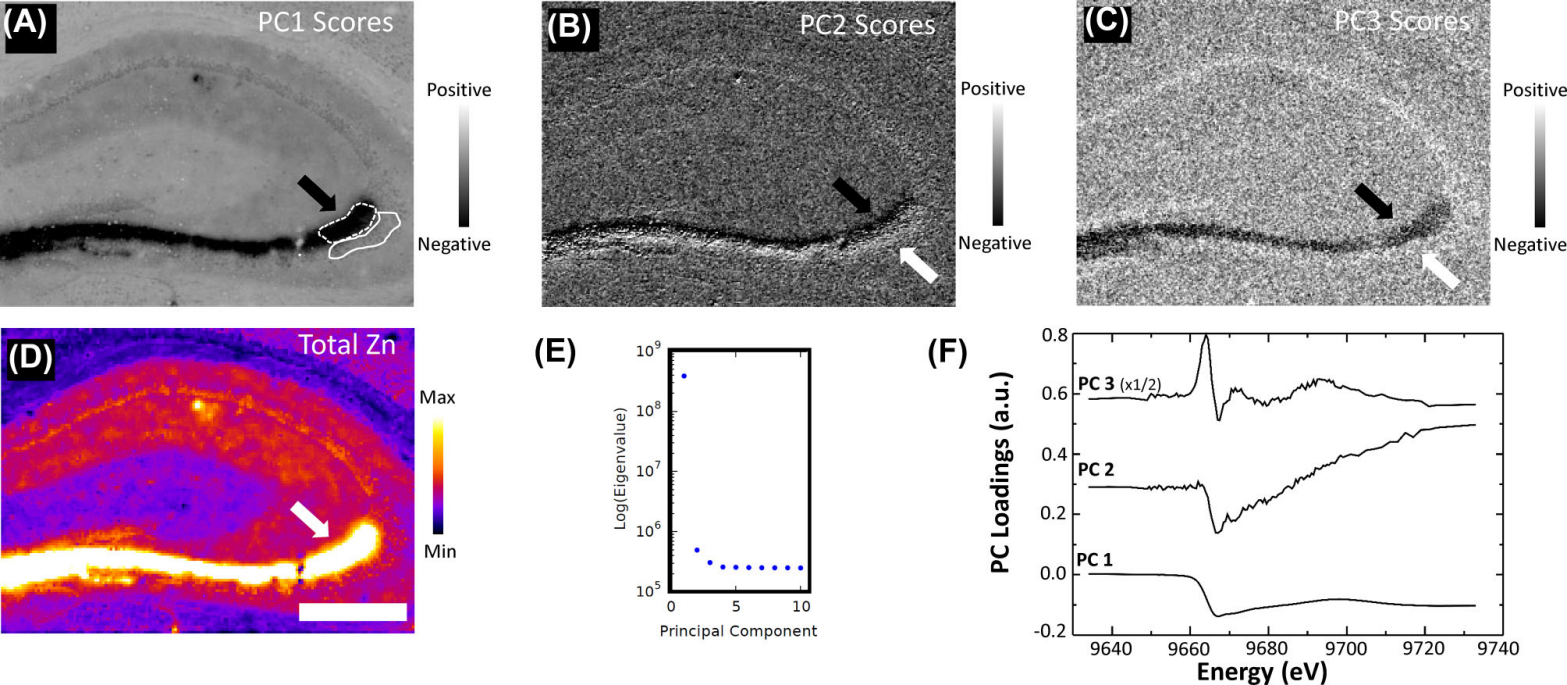
Principal component (PC) analysis provides an effective data reduction and exploration tool for XANES spectroscopic maps (using a representative example of an air-dried hippocampus section). (**A, B, C**) PC score images for PC1 (**A**), PC2 (**B**), and PC3 (**C**). The PC1 scores image reflects the total Zn image shown in panel (**D**), with most intense PC1 scores (intense negative scores) observed in regions of greatest Zn content, the CA3 mossy fibre region (black arrows). Relatively high Zn content is seen in the CA3 pyramidal neurons (white arrow), while the corpus callosum white matter (CC) displays low Zn content. PC2 and PC3 scores identify XANES spectroscopic variation within the CA3 region, with intense negative scores (black pixels) seen in the CA3 mossy fibre region (black arrow) and intense positive scores (white pixels) seen in the CA3 pyramidal payer (white arrow). (**E**) Log eigenvalue plot for the first 10 PC’s, which visually indicates 3 important PC’s. (**F**) PC loadings showing loadings plots for PC1-3, with PC2 and PC3 loadings have been offset vertically, and PC3 loadings scaled by ½, for clarity. The regions of interest that correspond to CA3 mossy fibres and CA3 pyramidal neurons, which were used for calculating average XANES spectra for linear combination fitting (Fig. 4, 5), are shown by white dashed line (CA3 mossy fibres) and white sold line (CA3 pyramidal neurons) in panel A. Scale bar = 500 µm.

The full-spectrum per pixel µXANES spectroscopic data sets were initially explored with principal component analysis (PCA), within the spectral microscopy data analysis package, Mantis. PCA provided a simple and time-effective approach to data-reduction to determine anatomical locations within the hippocampus that displayed variation in the Zn K-edge XANES spectrum. The PC score images of the first three PCs are shown in Fig. 2A—C, with the total Zn map shown for comparison in Fig. 2D. The eigenvalues are shown in Fig. 2E, which suggested 3 important PC’s in the data set. The corresponding PC1—3 loadings are shown in Fig. 2F. Not surprisingly the image produced from PC1 scores bears a strong resemblance to the total Zn map, and the loadings for PC1 resembles the average XANES spectrum. The PC1 scores image shows the most negative PC1 scores within the CA3 region of the hippocampus (the region containing highest total Zn content) [88] and more positive PC1 scores in the corpus callosum white matter (the region containing the lowest total Zn content). Of significant interest though, the PC2 and PC3 scores image show strong negative scores within the CA3 region containing mossy fibres, while the CA3 pyramidal neurons display strong positive scores. The general shape of the PC2 loadings shows similarity to the XANES spectrum of Zn^2+^ in the presence of excess phosphate (Fig. 1A), suggesting that there may be a substantial difference in Zn^2+^ phosphate coordination between the CA3 mossy fibres and CA3 pyramidal neurons (subsequently confirmed through least squares fitting, described below).

### µXANES spectroscopic mapping reveals unique distributions of Zn^2+^ speciation within the hippocampus

**Figure 3.**
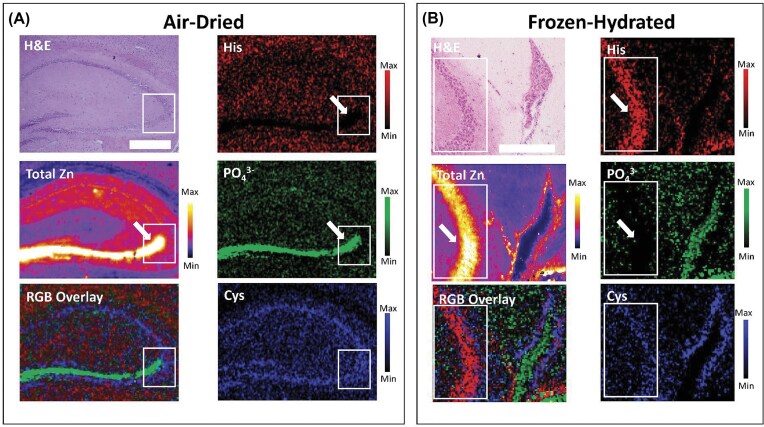
Chemically specific imaging of Zn^2+^ speciation with XANES spectroscopic mapping in air-dried and frozen hydrated tissues. (**A**) chemically specific Zn^2+^ images collected from air-dried mouse hippocampus tissue sections, compared to (**B**) chemically specific Zn^2+^ images collected from frozen-hydrated mouse hippocampus tissue sections. left column of images in both panels (**A**) and (**B**) show (top to bottom): (top) H&E histology of a serial tissue section (not the same section analysed with XANES spectroscopy); (middle) chemically specific Zn^2+^ imaging tri-colour overlay; and (bottom) total zn elemental map. right columns for both panel (**A**) and (**B**) show chemically specific Zn^2+^ images of (top to bottom): (top) Zn^2+^ speciation resembling Zn^2+^ in the presence of excess histidine; (middle) Zn^2+^ speciation resembling Zn^2+^ in the presence of excess PO_4_^3−^; and (bottom) Zn^2+^ speciation resembling Zn^2+^ in the presence of excess cysteine. white arrows highlight the zn rich ‘mossy fibre’ region which is enriched with Zn^2+^ coordinated to phosphates in air-dried tissue, but instead enriched in Zn^2+^ coordinated to histidine in frozen-hydrated tissue. Note: due to limited area of the cryostream, the field of view (sample map area) of samples analysed under cryostream was smaller than samples analysed at room temperature (to avoid parts of the sample moving outside of the crysotream during measurement and thawing). the white box in each of the panels shows the common anatomical region, containing CA3 pyramidal neurons and CA3 mossy fibres, between the two samples (air-dried, and frozen-hydrated) that are contained in this figure. Scale bar in A = 500 µm, and in B = 250 µm.

**Figure 4.**
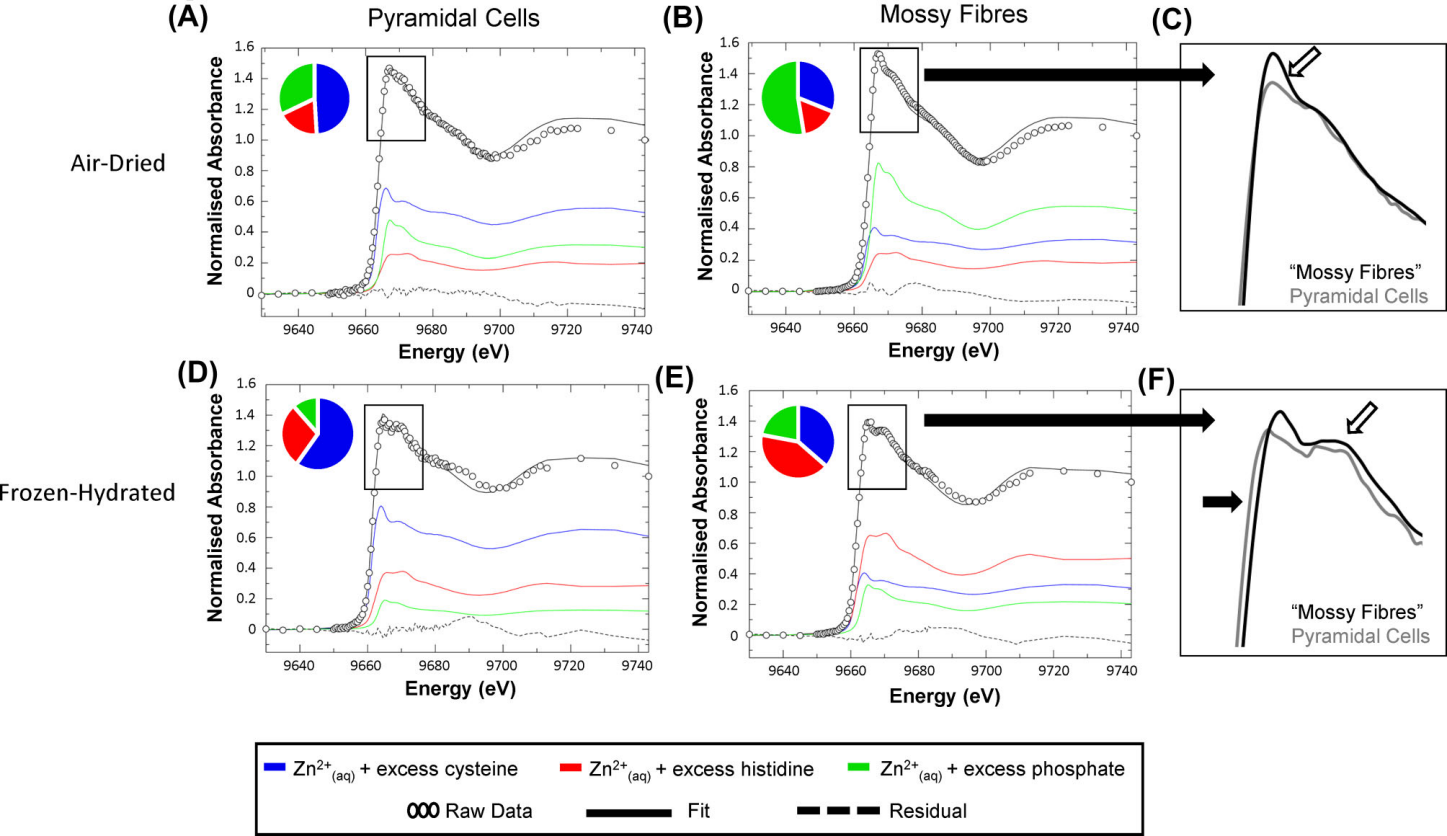
Representative examples of µXANES spectra corresponding to pyramidal neuron layer and ‘mossy fibre’ regions of interest, in air-dried and frozen-hydrated tissue sections, fit to the spectral library of standard solutions. (**A, B**) Fitting representative µXANES spectrum of (**A**) pyramidal cells and (**B**) ‘mossy fibres’ in air-dried tissue sections. (**C**) Zoomed in view of overlay of spectra from pyramidal cells and ‘mossy fibres’ in air-dried tissue, showing increased white line intensity (white arrow) in XANES spectra of mossy fibres (black trace) relative to pyramidal neurons (grey trace). (**D, E**) Fitting representative µXANES spectrum of (**D**) pyramidal cells and (**E**) ‘mossy fibres’ in frozen-hydrated tissue sections. (**F**) Zoomed in view of overlay of the spectra (real data, not fitted data) from ‘mossy fibres’ (black trace) and pyramidal cells (grey trace) in frozen-hydrated tissue, showing that ‘mossy fibres’ display a more pronounced higher energy shoulder (white arrow), while the rising edge of the white line is shifted to lower energies for spectra from pyramidal neuron layer (black arrow).

This study has applied for the first time a µXANES spectroscopic mapping protocol that generates ‘full-spectrum-per-pixel’ XANES stacks, which when de-convoluted using single value deconvolution in combination with our spectral library, to yield chemically specific images of Zn^2+^ speciation within the murine hippocampus (Fig. 3). As can be seen in Fig. 3, a striking differential distribution of Zn^2+^ speciation is observed within the hippocampus, in both dehydrated (air-dried) and frozen-hydrated tissue. The visual differences seen in the chemically specific Zn^2+^ images are confirmed by least square fitting of the average µXANES spectra of specific ROIs corresponding to the pyramidal neuron cell layer, and mossy fibres (Fig. 4).

**Figure 5.**
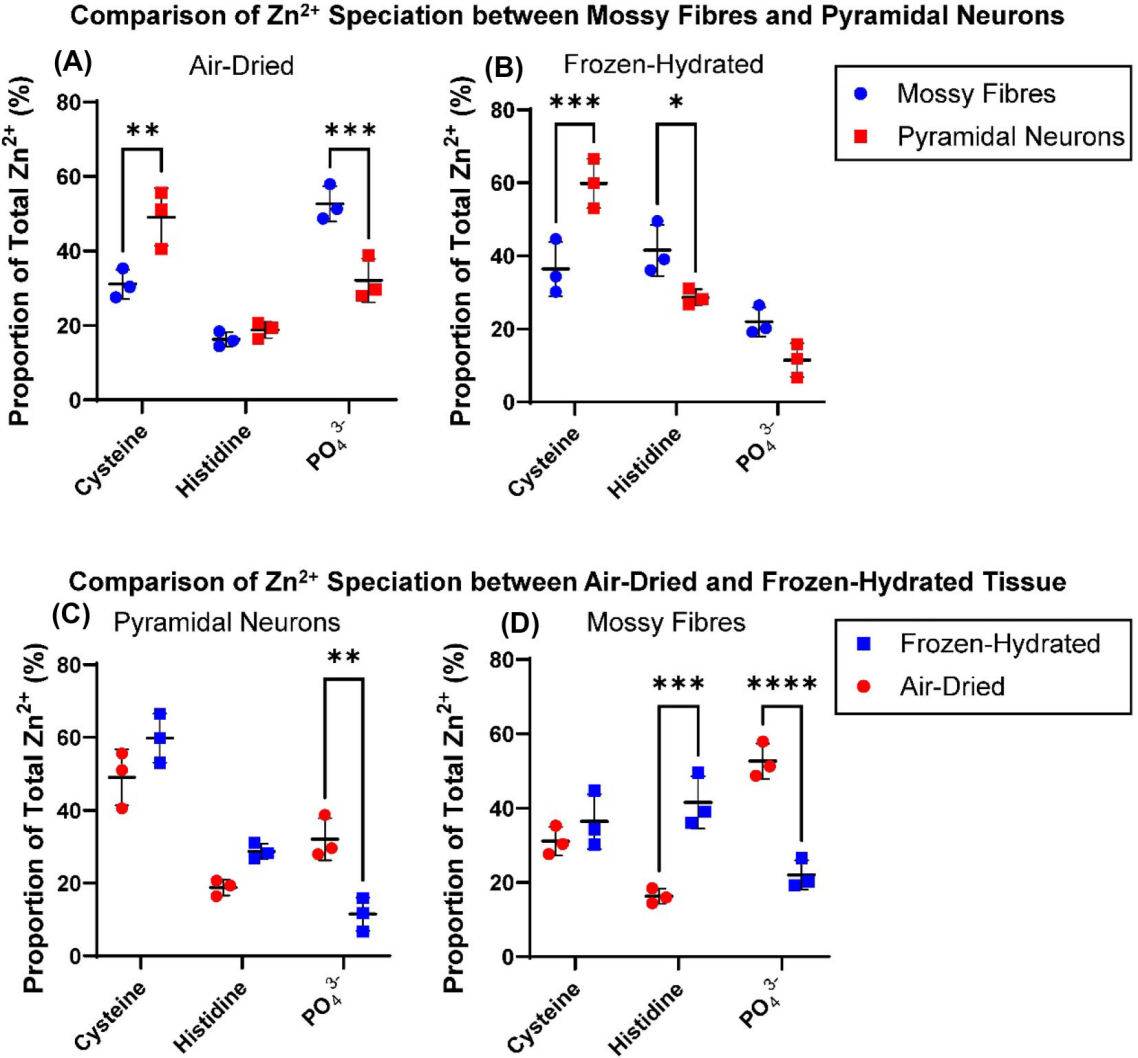
Results from statistical analysis performed on triplicate biological replicates comparing Zn^2+^ speciation in pyramidal neuron cell layer and ‘mossy fibres’, for air-dried and frozen-hydrated tissue sections. (**A**) comparison of Zn^2+^ speciation between pyramidal neurons and mossy fibres in air-dried tissue. (**B**) comparison of Zn^2+^ speciation between pyramidal neurons and mossy fibres in frozen-hydrated tissue. (**C**) comparison of Zn^2+^ speciation in pyramidal neurons between air-dried and frozen hydrated tissue. (**D**) comparison of Zn^2+^ speciation in ‘mossy fibres’ between air-dried and frozen hydrated tissue. * *P* < 0.05, ** *P* < 0.01, *** *P* < 0.001.

As can be seen in Fig. 4 (and confirmed with statistical analysis of replicates in Fig. 5), in air-dried tissue sections the hippocampal pyramidal neuron layer (in cornu ammonis sector 3, CA3) is enriched in Zn^2+^ coordinated through sulfur donors (e.g. coordination to thiol groups of cysteine residues). In contrast, chemically specific images of Zn^2+^ in a coordination environment resembling Zn^2+^—histidine complexes did not display localised enrichment in any specific hippocampal region in air-dried dehydrated tissues. Interestingly, a highly localised enrichment of Zn^2+^ consistent with coordination through phosphate groups was observed in the mossy fibre region (the mossy fibres are the location of major labile Zn^2+^ pools in the brain) [1, 11, 12, 41]. Intuitively, it is logical that the largest change in Zn^2+^ speciation that occurs during sample preparation (e.g. as discussed later in this manuscript, increased Zn^2+^—phosphate coordination during air-drying) is observed in the hippocampal region known to be most enriched in labile Zn^2+^ (the mossy fibre region).

Not unexpectedly, the chemically-specific maps of hydrated-frozen tissue sections analysed under cryogenic conditions, were visually different from air-dried tissue (Fig. 3). Specifically, in frozen-hydrated tissues there was significantly less Zn^2+^ in a coordination environment resembling coordination of Zn^2+^ through phosphates (relative to air-dried tissues, Fig. 3 –5). Indeed, in frozen-hydrated tissues the mossy fibres do not display localised enrichment of Zn^2+^ in an environment consistent with coordination to phosphates (Fig. 3 –5). Rather, the mossy fibre region in frozen-hydrated tissue displays increased abundance of Zn^2+^ in a coordination environment consistent with coordination to the imidazole N of histidine (Fig. 3 –5). The distribution of Zn^2+^ in a chemical form consistent with coordination through thiols in cysteine was largely consistent between air-dried and frozen-hydrated samples, with the pyramidal cell layer showing localised enrichment of thiol coordination for both air-dried dehydrated and frozen-hydrated tissues (Fig. 3).

The enrichment of the hippocampal pyramidal neuronal layer with Zn^2+^ speciation resembling coordination through four thiols groups, in both dehydrated and frozen-hydrated tissues, is in excellent agreement with the high metallothionein content of neurons (and eukaryotic cell cytoplasm in general) [89], and the abundance of Zn finger proteins in cell nuclei (which contain Zn^2+^ in tetrahedral coordination with four cysteine residues). It is also interesting to note the localised enrichment of Zn^2+^ coordination to thiols was also seen in images containing the lateral ventricles (e.g. Fig. 3B), as this brain region is characterised by high metallothionein protein and gene expression [90, 91]. As the brain lateral ventricles were not included in all samples imaged, statistical analysis was not undertaken.

### µXANES spectroscopic mapping indicates histidine residues are likely to be a dominant coordinating ligand of labile Zn^2+^ in the mossy fibres

Comparing the chemical form of Zn^2+^ observed in the hippocampal mossy fibres between the frozen-hydrated tissues and air-dried tissues provides important new insight into the chemical form of the labile Zn^2+^ pool. It is well known that the hippocampus and specifically the mossy fibres contain a substantial pool of labile Zn^2+^, which is critical to memory function and synaptic plasticity [3, 11, 92]. The chemical form of this pool of Zn^2+^ was however previously unknown. While the labile pool of Zn^2+^ is often referred to as ‘free’ Zn^2+^, in the absence of other ligands Zn^2+^ will exist as the octahedral hexaaqua complex and is not ‘free’ in the sense of a solvated alkali metal (e.g. Na^+^ or K^+^) [93]. Multiple studies have reported that due to the abundance of organic ligands in cells and tissues and the associated binding constants, it is highly unlikely that the labile Zn^2+^ pool would exist as the hexaaqua complex [93]. Indeed, spectroscopic studies by others have failed to observe evidence of the hexaaqua Zn^2+^ complex within Zn^2+^ enriched vesicles measured *in vitro* [67], and in this study we also did not observe spectroscopic evidence of the Zn^2+^ hexaaqua complex within the hippocampus. Our study has however observed a highly localised enrichment of Zn^2+^ within the mossy fibre region, where Zn^2+^ is in a coordination environment resembling Zn^2+^coordination through the N of the imidazole ring of histidine. Of significance, upon air-drying the tissue section there is a large change in Zn^2+^ speciation, with Zn^2+^—histidine coordination being replaced instead with Zn^2+^—phosphate coordination. We take the dramatic change in Zn^2+^ coordination upon air-drying of the tissue sections as strong evidence of the labile nature of the Zn^2+^—histidine coordination. Based on this, we propose that the labile Zn^2+^ pool exists *in vivo* as a coordination complex with histidine residues.

It is well established that the Zn^2+^ transport protein ZnT3 is critical to establishing/maintaining the labile Zn^2+^ pool within the CNS, especially the labile Zn^2+^ pool within the mossy fibres of the hippocampus [41, 94]. The exact binding sites of Zn^2+^ within ZnT3 haven’t being completely elucidated, however, the proteins contain several histidine rich domains, and most agree that the histidine rich domains of ZnT3 are the site of Zn^2+^ binding [95–97]. The results of chemically specific Zn^2+^ imaging in this study strongly support that the labile Zn^2+^ pool exists coordinated to histidine, possibly histidine residues of ZnT3 within the membrane of Zn^2+^ enriched vesicles. Given that the pK_a_ of histidine is 6.0, we speculate that our data supports existence of a pH shift (specifically reduction in pH) upon release of Zn-enriched vesicles during synaptic transmission, which weakens Zn^2+^—histidine coordination (due to protonation of the imidazole N), enabling Zn^2+^ release. This is in agreement with previous studies of binding mechanisms of ZnT5 [96].

### Sample preparation should be a crucial consideration when studying speciation of Zn in biological tissues

A key finding of this study is observation of the effects of sample preparation, specifically tissue dehydration, on Zn^2+^ speciation in brain tissue. Noticeably, air-dried tissue display a drastic increase in Zn^2+^ speciation resembling Zn^2+^ coordinated to phosphates (i.e. resembling the standard solution of Zn^2+^ in the presence of excess phosphate). The abundance of Zn^2+^—phosphate coordination in dehydrated tissues appears to be an artefact of sample preparation, as Zn^2+^—phosphate coordination was significantly less abundant in frozen-hydrated tissues where it accounted for 22% of Zn^2+^ on average relative to 53% on average in air-dried dehydrated tissues (Fig. 3 –5).

We account for the increase in Zn^2+^—phosphate coordination in air-dried dehydrated tissues through increased accessibility of phosphates to Zn^2+^ upon rupture of cell membranes during cryo-sectioning and consequent air-drying process (where the cytosol transitions from ice to a liquid prior to dehydration). During and following tissue sectioning and air-drying, labile Zn^2+^ and phosphates likely experience heightened intra-cellular and extra-cellular mobility as a consequence of cryo-sectioning induced membrane rupture. Although an apparent artefact of sample preparation, the drastic increase in phosphate coordination upon air-drying does however, highlight the labile nature of certain pools of Zn^2+^ in the CNS, and the propensity of labile Zn^2+^ to coordinate with phosphates if given opportunity.

### Limitations

This study has successfully imaged the major chemical forms of Zn^2+^ present in the hippocampus (Zn^2+^ coordinated to cysteine or histidine residues) however, there are obviously multiple other important Zn^2+^ coordination environments found across a range of Zn^2+^ metallo-proteins that were not detected. It should be noted that S/N levels with the µXANES spectra are substantially poorer than that obtained with a bulk XAS measurement (as described in Table 2), which may have contributed to a failure to detect less abundant (but biologically important) chemical forms of Zn^2+^. It is possible that in future work, imaging at increased spatial resolution, expansion of the standard solution and model compound library, or improved signal to noise in the data may enable additional forms of Zn^2+^ speciation to be imaged.

Although our data indicates that histidine coordination of Zn^2+^ is likely to exist in the labile Zn^2+^ pool, caution should be taken when interpreting these results. As has been shown by other authors, spectral mixtures of a range of Zn^2+^ thiol and imidazole nitrogen coordination environments (e.g. ZnS_4_, ZnS_3_N_1_, ZnS_2_N_2_, ZnS_1_N_3_, and ZnN_4_) can be adequately modelled by only two spectra (ZnS_4_ and ZnN_4_) [66]. Therefore, although our fitting indicates increased histidine coordination of the labile Zn^2+^, it doesn’t explicitly confirm whether coordination is exclusively histidine. Likewise, the results of our fitting do not differentiate between Zn^2+^ coordination of ‘free’ histidine or low molecular weight histidine-containing peptides, or histidine residues within proteins. Lastly, our spectral library is not exhaustive, and we can not claim with certainty that addition of another model spectrum (at this stage of an unknown compound) into the fitting procedure would still produce identical results.

## Conclusions

In summary, this study has expanded and characterised a XANES spectral library of standard solutions, which were then used to model and subsequently image Zn^2+^ speciation in murine brain tissue. The chemically specific images of Zn^2+^ speciation across an important brain region, the hippocampus, show clear differences in the chemical forms of Zn^2+^ present between neuron cell body layers and the ‘mossy fibre’ molecular layers. Sample preparation was shown to be a critical experimental consideration, with a drastic loss of Zn^2+^ coordination to histidine and subsequent increase in Zn^2+^ coordination to PO_4_^3−^ upon air-drying of tissue sections. This finding highlights that analysis of un-fixed, frozen-hydrated tissue sections is essential in order to preserve metal speciation as close as possible to the *in vivo* condition. Using the developed chemically specific Zn^2+^ imaging protocol, this study revealed fundamental new knowledge on the neurobiology of Zn^2+^; specifically, the pool of labile Zn^2+^ found in the ‘mossy fibres’ of the hippocampus has a coordination environment that is consistent with Zn^2+^ coordinated to histidine. Owing to the pH sensitive nature of histidine as a ligand, this observation provides new insight into a possible physiological pathway, through which an increase in pH could release labile Zn^2+^ from synaptic vesicles of ‘zincergic’ neurons in the ‘mossy fibre’ region. At this stage however, definitively proving the above pathway, and future work should now focus on demonstrating the biological mechanisms. Studies such as ‘organotypical’ living tissue slice models, which would enable pH manipulation, or analysis of Zn^2+^ speciation in additional animal models, such as ZnT3 KO mice, could prove highly informative.

## Supplementary Material

mfaf045_Supplemental_Files

## Data Availability

The data underlying this article will be shared on reasonable request to the corresponding author.
