# Supplementary material for: Imaging zinc speciation in the mouse hippocampus with µXANES Spectroscopic mapping
Source: Metallomics. 2026 Jan 2;18(1):mfaf045. doi: 10.1093/mtomcs/mfaf045 (PMC12859257; doi:10.1093/mtomcs/mfaf045)
Supplement: mfaf045_Supplemental_Files [file mfaf045_supplemental_files.zip › Suppl_Data.docx]

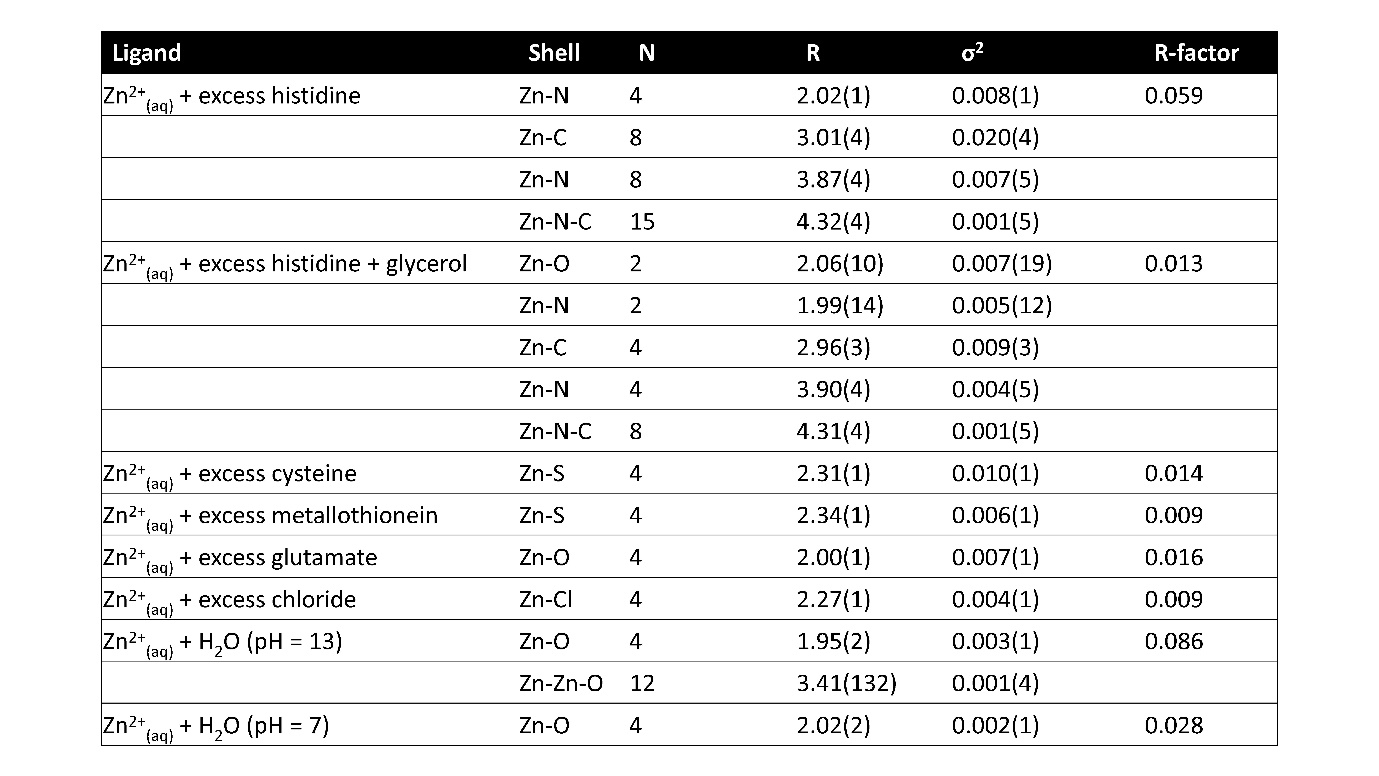


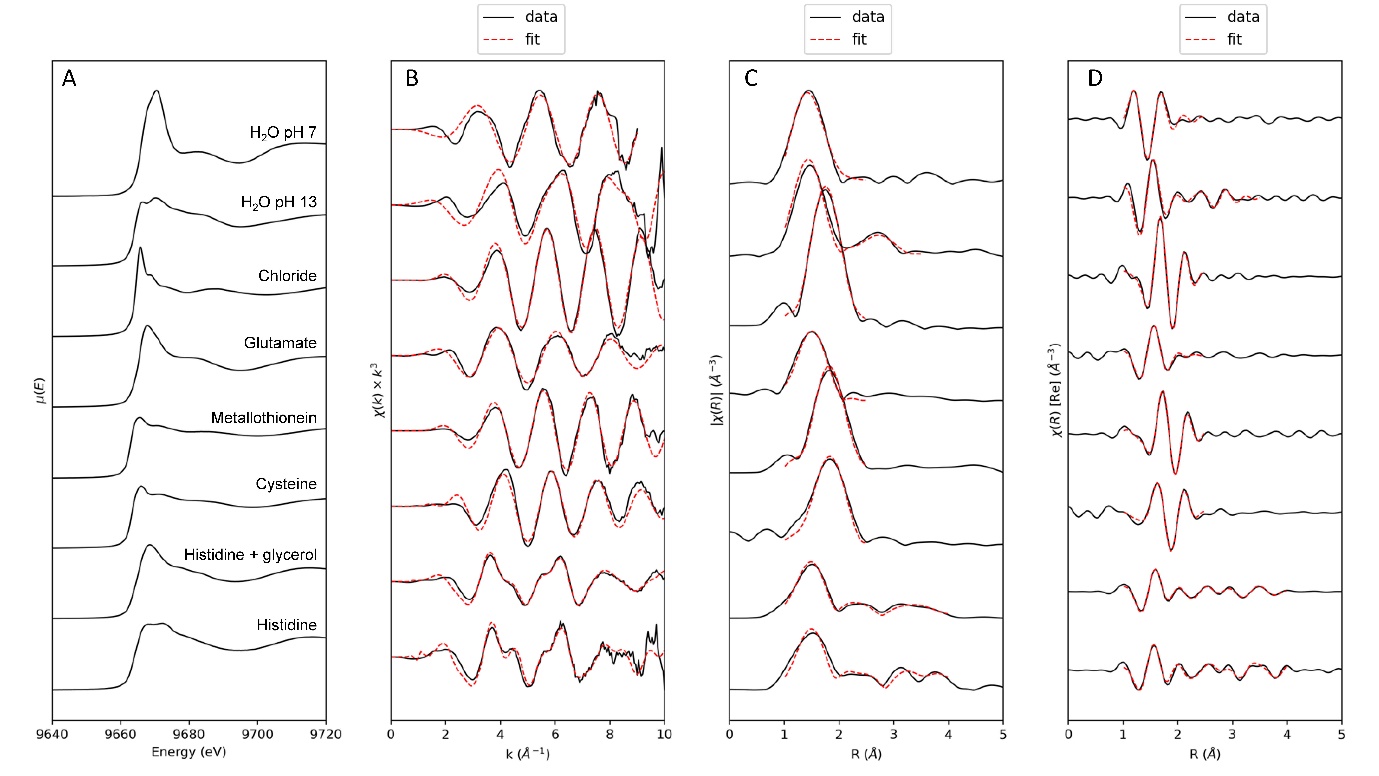


**Supporting Information Figure SI1:** XANES and EXAFS spectral characterisation of standard solutions. (**A**) Zn K-edge XANES spectra of standard solutions used for XANES spectral library. Dash lines indicate position of lowest energy white line feature for a standard solution in the library (9664.9 eV – metallothionein, and 9669.7 eV – hexaaqua Zn^2+^ +pH 7). (**B**) XANES spectra of standard solutions that were subsequently analysed with EXAFS spectroscopy. (**C**) Zn K-edge k^3^-weighted EXAFS spectra, (**D**) Fourier transform magnitude, and (**E**) Fourier transform real component with EXAFS fitting results for Zn^2+^ standard solutions. For the standard solution of Zn^2+^ in water at pH 13, a longer-range scattering peak is potentially observable, suggestive of a second shell Zn – Zn interaction. Although any interpretation of bonding environment beyond the first coordination shell is difficult due to the limited k-range of this data set, modelling of a Zn – Zn interaction, possibly suggesting the presence of a small amount of Zn(OH)_2_ precipitate in this sample, is shown in Supporting Information Figure 1.


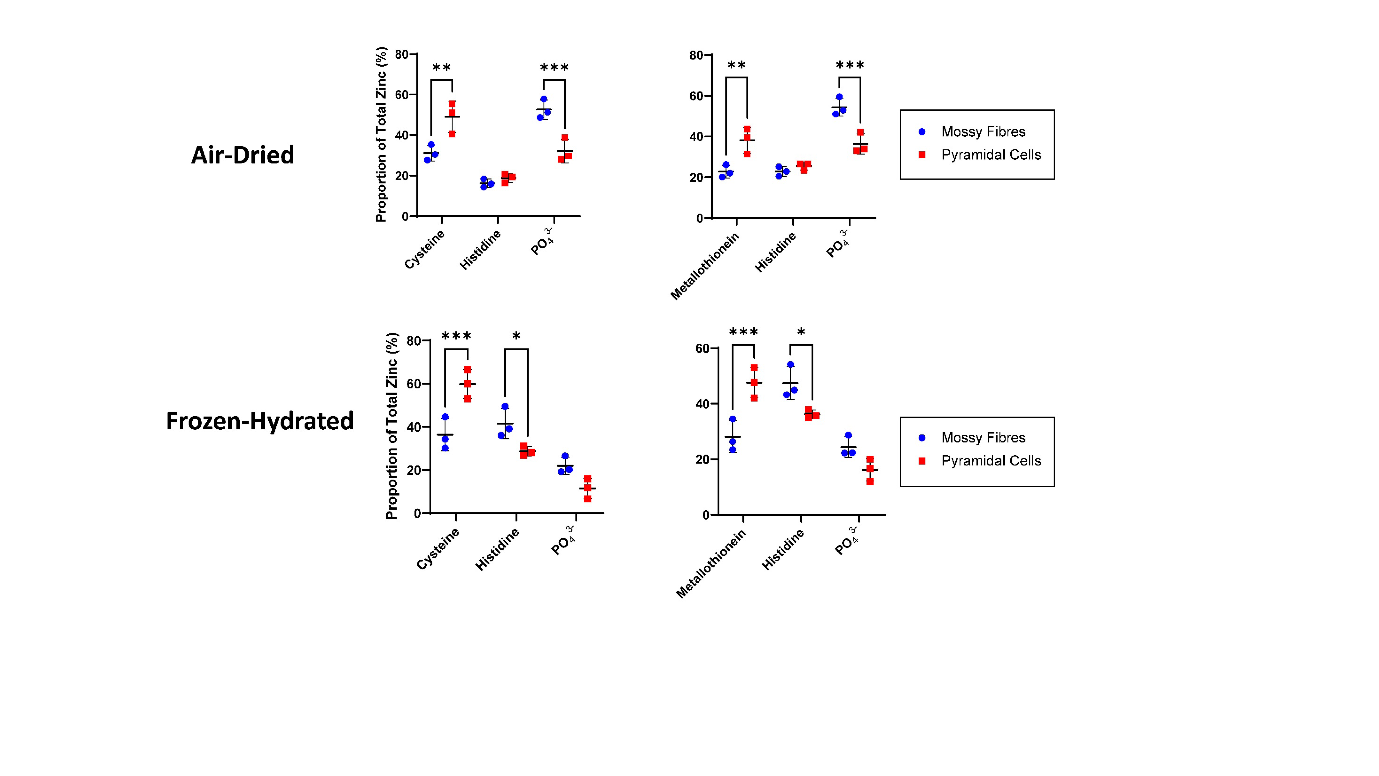


**Supporting Information Figure SI2**: Near identical fitting results and identical scientific conclusions are obtained if a metallothionein Zn K-edge XANES spectrum used in place of the Zn^2+^ + excess cysteine standard solution spectrum, for least squares fitting.

* *p* < 0.05, ** *p* < 0.01, *** *p* < 0.001
